# Rapid and structure-specific cellular uptake of selected steroids

**DOI:** 10.1371/journal.pone.0224081

**Published:** 2019-10-17

**Authors:** Jeffrey M. McManus, Kelsey Bohn, Mohammad Alyamani, Yoon-Mi Chung, Eric A. Klein, Nima Sharifi

**Affiliations:** 1 Department of Cancer Biology, Lerner Research Institute, Cleveland Clinic, Cleveland, Ohio, United States of America; 2 Department of Urology, Glickman Urological and Kidney Institute, Cleveland Clinic, Cleveland, Ohio, United States of America; 3 Department of Hematology and Oncology, Taussig Cancer Institute, Cleveland Clinic, Cleveland, Ohio, United States of America; Université de Genève, SWITZERLAND

## Abstract

Steroid hormones and their respective nuclear receptors are essential mediators in numerous physiologic and pathophysiologic processes, ranging from regulation of metabolism, immune function, and reproductive processes to the development of hormone-dependent cancers such as those of the breast and prostate. Because steroids must enter cells before activating nuclear receptors, understanding the mechanisms by which cellular uptake occurs is critical, yet a clear understanding of these mechanisms has been elusive. It is generally assumed that diffusion-driven uptake is similar across various steroids whereas an elevated cellular concentration is thought to reflect active uptake, but these assumptions have not been directly tested. Here we show that intact cells rapidly accumulate free steroids to markedly elevated concentrations. This effect varies widely depending on steroid structure; more lipophilic steroids reach more elevated concentrations. Strong preferences exist for 3β-OH, Δ^5^-steroids vs. 3-keto, Δ^4^-structural features and for progestogens vs. androgens. Surprisingly, steroid-structure-specific preferences do not require cell viability, implying a passive mechanism, and occur across cells derived from multiple tissue types. Physiologic relevance is suggested by structure-specific preferences in human prostate tissue compared with serum. On the other hand, the presence of serum proteins in vitro blocks much, but not all, of the passive accumulation, while still permitting a substantial amount of active accumulation for certain steroids. Our findings suggest that both passive and active uptake mechanisms make important contributions to the cellular steroid uptake process. The role of passive, lipophilicity-driven accumulation has previously been largely unappreciated, and its existence provides important context to studies on steroid transport and action both in vitro and in vivo.

## Introduction

Steroid hormones and their respective nuclear receptors are important mediators in normal physiology and pathophysiologic processes. Steroids are synthesized from cholesterol through a series of enzymatic reactions, with major structural modifications that include conversion from the 3β-OH, Δ^5^-structure (that is shared with cholesterol) to 3-keto, Δ^4^-steroids by 3β-hydroxysteroid dehydrogenase (3β-HSD) and conversion from progestogens to androgens by 17α-hydroxylase/17,20-lyase (CYP17A1) [1, 2]. Steroid hormones act by binding to their cognate nuclear receptors, such as androgen receptor (AR), estrogen receptors (ERα and ERβ), glucocorticoid receptor (GR), and progesterone receptor (PR), resulting in ligand-stimulated transcription of target genes [3]. These receptors have key roles in development and physiology, ranging from regulation of numerous aspects of metabolism and immune function by GR, to control of female reproductive processes by PR, to normal male development by AR. Steroid receptors are also involved in various disease states including hormone-dependent cancers such as those of the breast and prostate, driven by ER and AR, respectively [4, 5]. Steroids are either directly secreted into the circulation in their active form, or are secreted as precursors and converted by steroidogenic enzymes in peripheral target tissues to receptor agonists. For example, dehydroepiandrosterone (DHEA) is secreted from the human adrenal gland and converted in peripheral tissues to biologically active androgens and estrogens [6].

Regardless of whether they are precursor steroids or are secreted in the form that directly stimulates signaling, steroids must enter cells before activating nuclear receptors. By what mechanisms does this occur? Free steroids (e.g. testosterone, estradiol, DHEA, etc.) are lipophilic molecules that are often presumed to freely diffuse across cell membranes. However, in vivo a large proportion of steroid molecules in serum are not free but are bound to proteins such as albumin and sex hormone binding globulin (SHBG) [7]. According to the free hormone hypothesis, the pool of biologically active hormones available to tissue consists of the free, not the protein-bound, hormones. This question has not been conclusively resolved [8, 9] as it has been suggested that the protein-bound pool of hormones is also available for exchange into cells [10] and that steroids, particularly conjugated steroids, might utilize transmembrane transporters for cellular uptake [11]. Research going back several decades has provided at times seemingly contradictory evidence for passive uptake, active uptake, or both, of steroid as well as thyroid hormones [12, 13]. The underlying general assumption for most frameworks of steroid action is that passive diffusion of unconjugated steroids is comparable among different steroids, including both precursor steroids and steroids that directly bind their respective nuclear receptors [14, 15], and yet even this view has been recently challenged as it has been suggested that in some systems steroids may be unable to passively diffuse into cells and may require active transport [16, 17].

A fundamental question when considering these issues is this: when cells are exposed to a given concentration of free (unbound) steroid, what is the resulting intracellular concentration? Because steroids are thought to freely diffuse across cell membranes, one might assume, in the absence of active mechanisms, that the two concentrations would be comparable. On the other hand, there is some recognition in the endocrine disruptors field that environmental steroids accumulate at heightened concentrations in aquatic organisms and this bioaccumulation may depend on steroid molecules’ physicochemical properties, particularly their degree of lipophilicity [18–20]. To our knowledge, these principles have not received attention in the steroid physiology field, and the cell’s natural predilection for structure-specific steroid uptake has not been studied in a systematic manner.

## Materials and methods

All patient samples were obtained using written informed consent with a Cleveland Clinic Institutional Review Board-approved protocol (IRB5923).

### Cell lines

LNCaP prostate cancer cells were purchased from the American Type Culture Collection (Manassas, VA) and maintained in RPMI-1640 with 10% FBS. JEG-3 placental choriocarcinoma cells were purchased from the American Type Culture Collection and maintained in Eagle's Minimum Essential Medium with 10% FBS. HMC-1-8 breast cancer cells [21] were provided by M. Abazeed and maintained in RPMI-1640 with 10% FBS.

### Steroid uptake

Cells (~1,000,000 cells per well) were plated in 6-well plates coated with poly-L-ornithine. The day after seeding, medium was replaced with 800 μL serum-free medium and cells were treated with [^3^H]-labeled steroids (~100,000 cpm) (PerkinElmer, Waltham, MA). After one-hour incubation at 37°C, media were removed and transferred to 2 mL microcentrifuge tubes (USA Scientific, Ocala, FL). Cells were washed once with D-PBS, then scraped into 800 μL D-PBS and transferred to 2 mL microcentrifuge tubes. Cell samples were subjected to 3x freeze-thaw cycles to lyse cells. We observed that some fraction of the steroids in samples adhered to plastic tubes, so 700 μL 1:1 ethyl acetate:isooctane (1:1) was added to each media and cell sample, which prevented this adhesion. Total contents of each microcentrifuge tube were then transferred to scintillation vials containing Liquiscint scintillation fluid (National Diagnostics, Atlanta, GA). Total radioactive signal in each sample was assayed by LS 6000IC scintillation counter (Beckman Coulter, Brea, CA). In each experiment, cells from two untreated wells were scraped into D-PBS and volumes of cell pellets were measured using PCV packed cell volume tubes (TPP Techno Plastic Products AG, Trasadingen, Switzerland). The volumes of the pellets from the two wells were averaged and the average volume was used for cellular concentration (counts/μL) calculations from that experiment. Typical volumes of pellets were approximately 3 to 5 μL. All experiments were performed with biological duplicates and repeated independently at least twice. In these experiments specifically for JEG-3 cells treated with pregnenolone, [^3^H]-labeled pregnenolone was supplemented with 10 μM unlabeled pregnenolone because we observed that rapid conversion by enzyme 3βHSD of pregnenolone to progesterone in JEG-3 cells (see Results section) resulted in decreased cellular content of [^3^H]-labeled steroid after one hour, as progesterone has much less cellular affinity than pregnenolone. 10 μM pregnenolone saturated this 3βHSD-catalyzed reaction and enabled more accurate measurement of pregnenolone uptake after one hour. For this purpose of saturating the 3βHSD-catalyzed reaction, it was not necessary to add a saturating concentration of pregnenolone for the experiments in other cell lines, as LNCaP cells exhibited little conversion of pregnenolone in one hour and HMC-1-8 cells exhibited none (data not shown). Note that the effect on pregnenolone uptake of adding a saturating concentration of pregnenolone was, however, tested in LNCaP cells in a separate experiment (see Results section).

Experiments assaying uptake by mass spectrometry were performed in the same way as normal steroid uptake experiments except that 100 nM unlabeled steroids (Steraloids, Newport, RI) were used in lieu of [^3^H]-labeled steroids. After media and cell samples were collected and cells were lysed via 3x freeze-thaw cycles, 10 μL internal standard [50 ng/mL, AD-^13^C_3_] was added to each sample and samples were transferred to glass test tubes for liquid-liquid extraction. 2 mL methyl tert-butyl ether (MTBE) was added to each tube; tubes were vortexed for 5 minutes and centrifuged (1500 g, 5 min, 4°C) and the MTBE layer was collected and then dried under nitrogen gas and reconstituted in 120 μL 50% methanol/water (v/v). The extracted steroids were quantified using liquid chromatography tandem mass spectrometry (LC-MS/MS). Briefly, the extracted steroids were injected onto a Shimadzu Nexera 2 UHPLC system (Shimadzu Corporation, Kyoto, Japan), and the steroids were separated on a C18 column (Zorbax Eclipse Plus C_18_ column, 150 mm x 2.1 mm, 3.5 μm, Agilent, Santa Clara, CA) using a gradient starting from 20% solvent B [acetonitrile/methanol (90/10, v/v) containing 0.2% formic acid] for 4 min and then to 75% solvent B for 10 min, followed by 95% solvent B for 3 min. The steroids were quantified on a Qtrap 5500 mass spectrometer (AB Sciex, Framingham, MA) using ESI in positive ion mode and multiple reaction monitoring (MRM) using characteristic parent → daughter ion transitions for the specific molecular species monitored. Androstene-3,17-dione-2,3,4-^13^C_3_ (Cerilliant, Round Rock, TX) was used as an internal standard for calibration of steroids in each sample. Data acquisition and processing were performed using MultiQuant (AB Sciex; version 3.0.1). The peak area ratio of the analyte over the internal standard was used for quantification. Each sample run included a calibration curve with standards for data quantification using the analyte/internal standard peak area ratio.

For parallel live and dead cell experiments, cells (~1,500,000 cells per tube) were aliquoted into 1.5 mL microcentrifuge tubes (Thermo Fisher Scientific, Waltham, MA) in 1 mL serum-free media. For the dead cell condition, tubes were heated to 90–95°C for 15 min. Cells were confirmed to be dead by trypan blue assay using a Countess II FL Automated Cell Counter (Thermo Fisher Scientific). Live and dead cells were then treated in parallel with [^3^H]-labeled steroids (~100,000 cpm) and incubated for one hour at 37°C on tube rotators. In an additional set of experiments, cells were treated with 2 nM [^3^H]-labeled steroids with or without the addition of 1 μM unlabeled steroids. Cells were pelleted by 5 min. 250 g centrifugation in an Allegra 6R centrifuge (Beckman-Coulter). Under this protocol, dead HMC-1-8 cells, unlike live HMC-1-8 cells and live or dead LNCaP and JEG-3 cells, did not form good pellets, so HMC-1-8 cells were not included in this experiment. After pelleting, media were removed from tubes and transferred to 2.0 mL microcentrifuge tubes. Cell pellets were collected in 800 μL D-PBS and transferred to 2.0 mL microcentrifuge tubes. Cell samples were subjected to 3x freeze-thaw cycles to lyse cells. 800 μL 1:1 ethyl acetate:isooctane (1:1) was added to each media and cell sample to prevent steroid adhesion to tubes. Total contents of each microcentrifuge tube were then transferred to scintillation vials containing scintillation fluid and total radioactive signal in each sample was assayed by scintillation counter. In each experiment, volumes of cell pellets from untreated tubes (two tubes each of live and dead cells) were measured using PCV tubes.

Experiments testing uptake in the presence of serum were performed in the same way as normal steroid uptake experiments except that culture media were replaced with human serum (Sigma-Aldrich, St. Louis, MO) or charcoal-stripped fetal bovine serum (Gemini, West Sacramento, CA). Steroid concentrations for these experiments were 2 nM [^3^H]-labeled steroid with or without the addition of 1 μM unlabeled steroid. Additionally, because we observed that serum did not wash out of dishes as readily as culture media, parallel experiments were performed with no cells and a correction was applied to the results with cells by subtracting the steroid content of the residual serum in the control experiments from the steroid content of the cell samples.

### Calculations

Each experimental treatment resulted in corresponding measurements of total cellular signal and total media signal. The cellular signal was divided by the cell volume from PCV tube measurement to obtain the cellular concentration (counts/μL). The cellular signal and media signal were added together and divided by the media volume to obtain the original media concentration upon treatment (counts/μL). The cellular concentration was divided by the original media concentration to obtain the ratio reported in the graphs throughout the results section.

### Time course experiments

The uptake of DHEA, pregnenolone, progesterone, and Δ4-androstenedione (AD) was measured in LNCaP cells as previously described with minor modifications [22]. Briefly, cells (4.5x10^5^ cells per well) were plated in poly-L-ornithine coated 12-well plates with 10% charcoal stripped fetal bovine serum (CSS) phenol red-free RPMI and incubated for 48 h at 37°C. Cells were then incubated with 10 nM [^3^H]-steroid in transport buffer (25 mM HEPES, 125 mM NaCl, 4.8 mM KCl, 5.6 mM D-glucose, 1.2 mM CaCl_2_, 1.2 mM KH_2_PO_4_, and 1.2 mM MgSO_4_, pH 7.4) at 37°C for the indicated time points. At each time point, the media from the wells was collected, and the cells were washed with ice cold transport buffer. Cells were lysed with 1% TritonX-100 in PBS, collected, and protein concentration determined by BCA assay. Samples were added to 3 mL Liquiscint, and radioactivity was measured on a liquid scintillation counter. Results were graphed as (pmol intracellular steroid/pmol total steroid)/protein concentration in units of μL/mg using GraphPad Prism 5 (GraphPad Software, San Diego, CA).

### Patient samples

All patient samples were obtained using an Institutional Review Board-approved protocol. Matching serum and tissue samples from patients with localized prostate cancer undergoing radical prostatectomy were subject to liquid chromatography tandem mass spectrometry analysis (LC-MS/MS). Steroids (pregnenolone, progesterone, DHEA, and AD) and their corresponding internal standards (d4-pregnenolone, d9-progesterone, d2-DHEA, and ^13^C-AD) were extracted from the tissue samples after homogenization with methanol followed by solid phase extraction. Serum samples (100 μL) were subject to liquid liquid extraction; the steroids and the internal standards were extracted with MTBE evaporated to dryness under N_2_ then reconstituted with 200 μL of 50% methanol. Steroids and their corresponding internal standards were ionized using electrospray ionization in positive mode on an AB Sciex 5500 Qtrap coupled with Shimadzu Nexera UPLC station.

### Fractionation experiments

Subcellular fractionation was performed using a modified version of a previously described protocol [23, 24]. LNCaP cells were seeded on 10 cm plates. After cells reached confluency, growth media were replaced with serum-free media and cells were treated with 1 μM unlabeled pregnenolone, progesterone, DHEA, or AD and incubated for 1 hour at 37°C. Media samples were collected, remaining media were removed, and cells were scraped into 500 μL fractionation buffer (20 mM HEPES, 10 mM KCl, 2 mM MgCl_2_, 1 mM EDTA, 1 mM EGTA, pH 7.4; just before use, 1 mM DTT and 1 Pierce Protease Inhibitor table (Thermo Fisher Scientific) were added to 50 mL buffer). Cells were incubated on ice for 15 minutes, then lysed by using a 1 mL syringe to pass through a 27 gauge needle 10 times, then left on ice for an additional 20 minutes. Lysate was then centrifuged for 5 min at 720 g and 4°C on a 5424R centrifuge (Eppendorf, Hamburg, Germany) and supernatant was collected and transferred to a fresh tube. This supernatant was centrifuged for 5 min at 10,000 g and 4°C, the new supernatant was transferred to a 14 x 25 mm Quick Seal centrifuge tube (Beckman Coulter), and additional fractionation buffer was added to fill the tube (total volume 3.5 mL). Samples were then centrifuged for 1 h at 100,000 g and 4°C on an Ultima L-100 XP ultracentrifuge with SW 41Ti rotor (Beckman Coulter). The supernatant was removed, forming the cytosol fraction, and the pellet, forming the membrane fraction, was collected in 550 μL fractionation buffer. Samples were analyzed by mass spectrometry as described above.

## Results

### Cells derived from several tissue types accumulate steroids at high concentrations and uniformly have preferences for 3β-OH, Δ^5^-steroids over 3-keto, Δ^4^-steroids and progestogens over androgens

**Fig 1:**
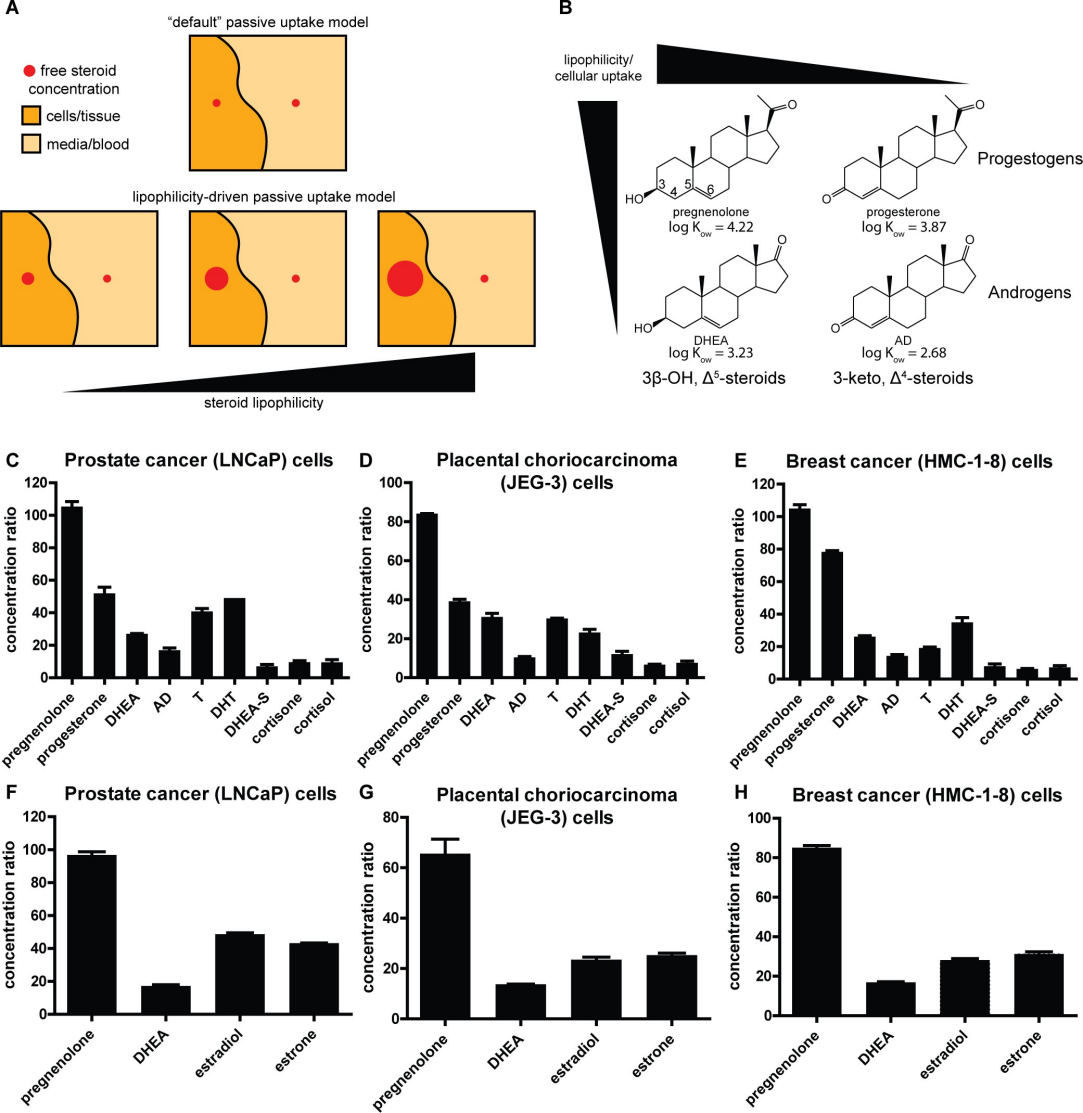
Cells derived from several tissue types accumulate steroids at high concentrations and uniformly have a preference for 3β-OH, Δ^5^-steroids over 3-keto, Δ^4^-steroids and progestogens over androgens. **(A)** Schematic representation of cellular steroid uptake. In a model of uptake driven solely by passive diffusion and disregarding lipophilicity-driven bioaccumulation (top panel), concentrations of free steroids inside and outside cells would be equivalent. When lipophilicity-driven bioaccumulation is taken into account, steroid concentrations in cells or tissue can become greater than those in media or blood, and to a much greater extent for more lipophilic than for less lipophilic steroids. **(B)** A specific example of the concept schematically represented in **(A)**: degree of steroid uptake depends on molecular structure; for example, progestogens (e.g. pregnenolone and progesterone) are more lipophilic and are preferentially taken up more than corresponding androgens (e.g. DHEA and AD) and 3β-OH, Δ^5^-steroids (e.g. pregnenolone and DHEA) are more lipophilic and are preferentially taken up more than corresponding 3-keto, Δ^4^-steroids (e.g. progesterone and AD). For reference, carbons 3, 4, 5, and 6 are labeled on the pregnenolone structure. Note the hydroxyl group at position 3 and double bond between carbons 5 and 6 on the 3β-OH, Δ^5^-steroids and the carbonyl group at position 3 and double bond between carbons 4 and 5 on the 3-keto, Δ^4^-steroids. Log K_ow_ values, a measure of lipophilicity [25], are shown for the four steroids. **(C-E)** Ratios of cellular concentrations to original treatment concentrations in culture media for nine different steroids in **(C)** prostate cancer (LNCaP) cells, **(D)** placental choriocarcinoma (JEG-3) cells, and **(E)** breast cancer (HMC-1-8) cells. **(F-H)** Ratios of cellular concentrations to original treatment concentrations in culture media for four different steroids in **(F)** prostate cancer (LNCaP) cells, **(G)** placental choriocarcinoma (JEG-3) cells, and **(H)** breast cancer (HMC-1-8) cells. All graphs show mean ± SD from one representative experiment with biological duplicates and all experiments were performed at least twice. For all graphs, the uptake of pregnenolone was greater than all other steroids (p < 0.001, Tukey’s multiple comparison test after one-way ANOVA). Additionally, uptake of progesterone was greater than AD (p < 0.001) and uptake of DHEA was greater than AD (C: p = 0.01; D-E: p < 0.001).

To test cells’ tendency to take up steroids (**Fig 1A**) and the effects of steroid structural differences on uptake (**Fig 1B**), human cell lines derived from prostate, breast, and placenta were treated in vitro with nine different [^3^H]-labeled steroids in parallel: pregnenolone, progesterone, DHEA, Δ^4^-androstenedione (AD), testosterone (T), dihydrotestosterone (DHT), DHEA sulfate (DHEA-S), cortisol, and cortisone (**Fig 1C–1E**). Uptake of estradiol and estrone was tested in a second set of experiments, in parallel with pregnenolone and DHEA to aid in comparison to the initial set of experiments (**Fig 1F–1H**). Although there were minor differences between cell lines, the overall uptake trends were surprisingly consistent across the three cell lines. All tested steroids had substantially greater concentrations in the cells after 1 hour than in the culture media. Our major observation was that pregnenolone reached the highest concentrations of any steroid, with concentrations up to roughly 100 times the original treatment concentrations in the culture media, and that cells have strong preferences for 3β-OH, Δ^5^-steroids (i.e., pregnenolone and DHEA) vs. 3-keto, Δ^4^-structural features (i.e., progesterone and AD) and for progestogens vs. androgens.

### Cellular preferences for progestogens and 3β-OH, Δ^5^-steroids are independent of cell viability

**Fig 2:**
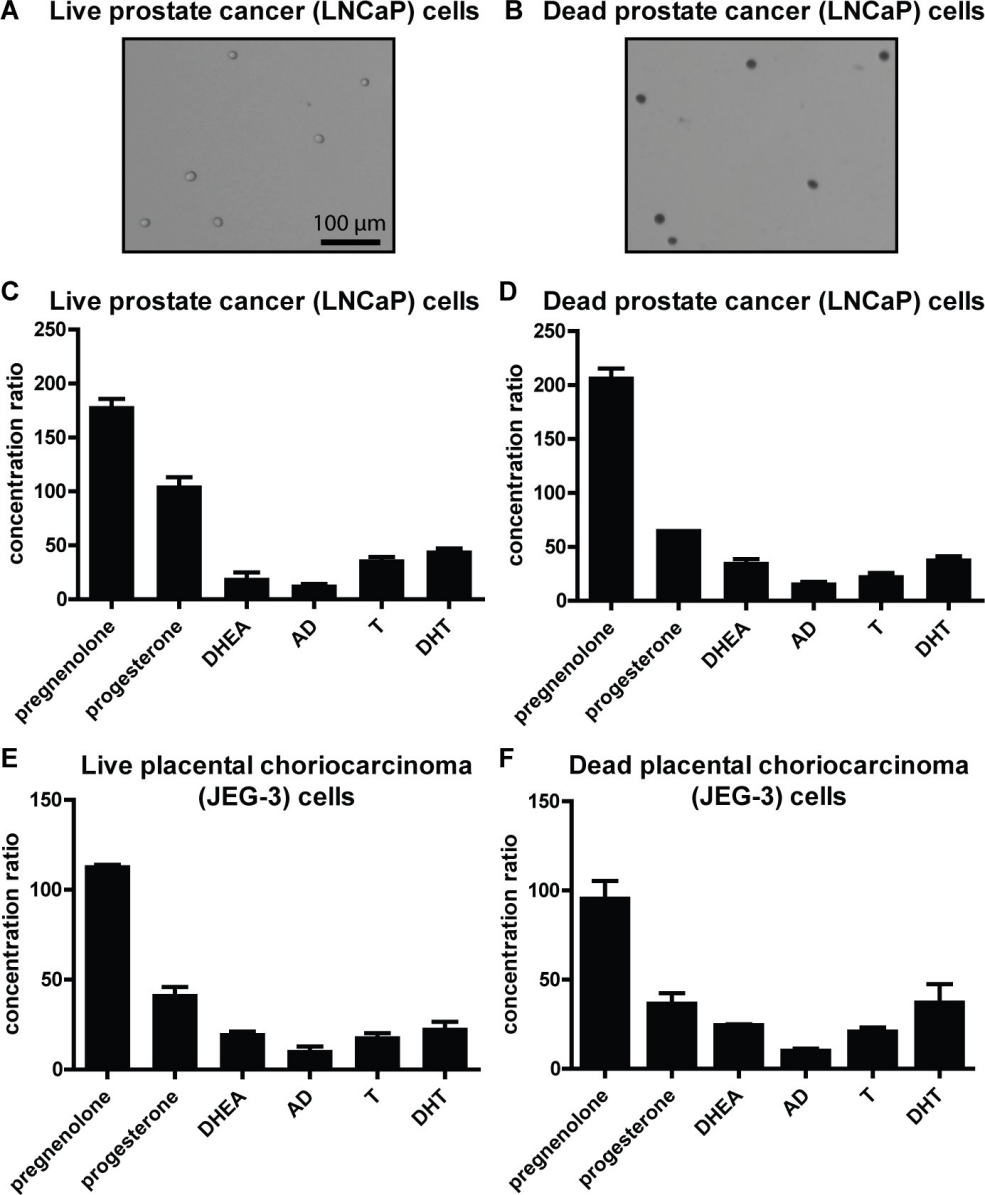
Cellular preferences for progestogens and 3β-OH, Δ^5^-steroids are independent of cell viability. **(A-B)** Live **(A)** and heat-killed **(B)** LNCaP cells exposed to trypan blue stain. Note the staining of the heat-killed cells, and note also that the live and dead cells are similar from a gross morphological standpoint. **(C-D)** Ratios of cellular concentrations to original treatment concentrations in culture media for six different steroids in live **(C)** and dead **(D)** LNCaP cells incubated in tubes. **(E-F)** Ratios of cellular concentrations to original treatment concentrations in culture media for six different steroids in live **(E)** and dead **(F)** JEG-3 cells incubated in tubes. All graphs show mean ± SD from one representative experiment with biological duplicates and all experiments were performed at least twice.

Our finding that cells consistently have a large capacity for steroid uptake that differs greatly in magnitude for different steroids raises the question of the nature of the uptake mechanism. To test whether the uptake involves active cellular processes, we assayed uptake of six [^3^H]-labeled steroids (pregnenolone, progesterone, DHEA, AD, testosterone, and DHT) in cells that were heat killed prior to steroid treatment. In these experiments, cells in suspension in microcentrifuge tubes were killed by heating to 90–95°C, removed from the heat, and then treated with steroids in parallel to live cells that underwent the same procedure except for the heating. Cell death of the heat-killed cells was verified by trypan blue assay (**Fig 2A and 2B**). Because heating cells to 90–95°C would result in both permeabilization of membranes [26] and denaturation of proteins [27], under this condition steroids can freely move into and out of cells and any elevated cellular concentration should be the result of passive, not active, processes. After incubation with steroids, both live and dead cells were pelleted and the steroid content of the media and cell pellets was measured. The results were striking: the uptake magnitudes were generally similar in dead cells as in parallel treated live cells (**Fig 2C–2F**). Crucially, the existence of elevated steroid concentrations in both live and dead cells implies not only that steroids can enter cells regardless of cell viability, but also that some force independent of any active cellular processes (i.e. active transport, active binding to intracellular proteins, or both) causes steroids to be retained in the cells at greatly elevated concentrations and in a structure-specific manner. To verify the findings using an alternate experimental method, uptake of unlabeled steroids (pregnenolone, DHEA, AD) in both live and dead cells was assayed by mass spectrometry and similar trends were observed as with [^3^H]-labeled steroids (**S1 Fig**). These results support the conclusion that a passive mechanism drives cellular preferences for progestogen and 3β-OH, Δ^5^-steroid uptake.

### Live vs. dead cell uptake comparisons and uptake saturation both reveal contributions of active and passive accumulation

**Fig 3:**
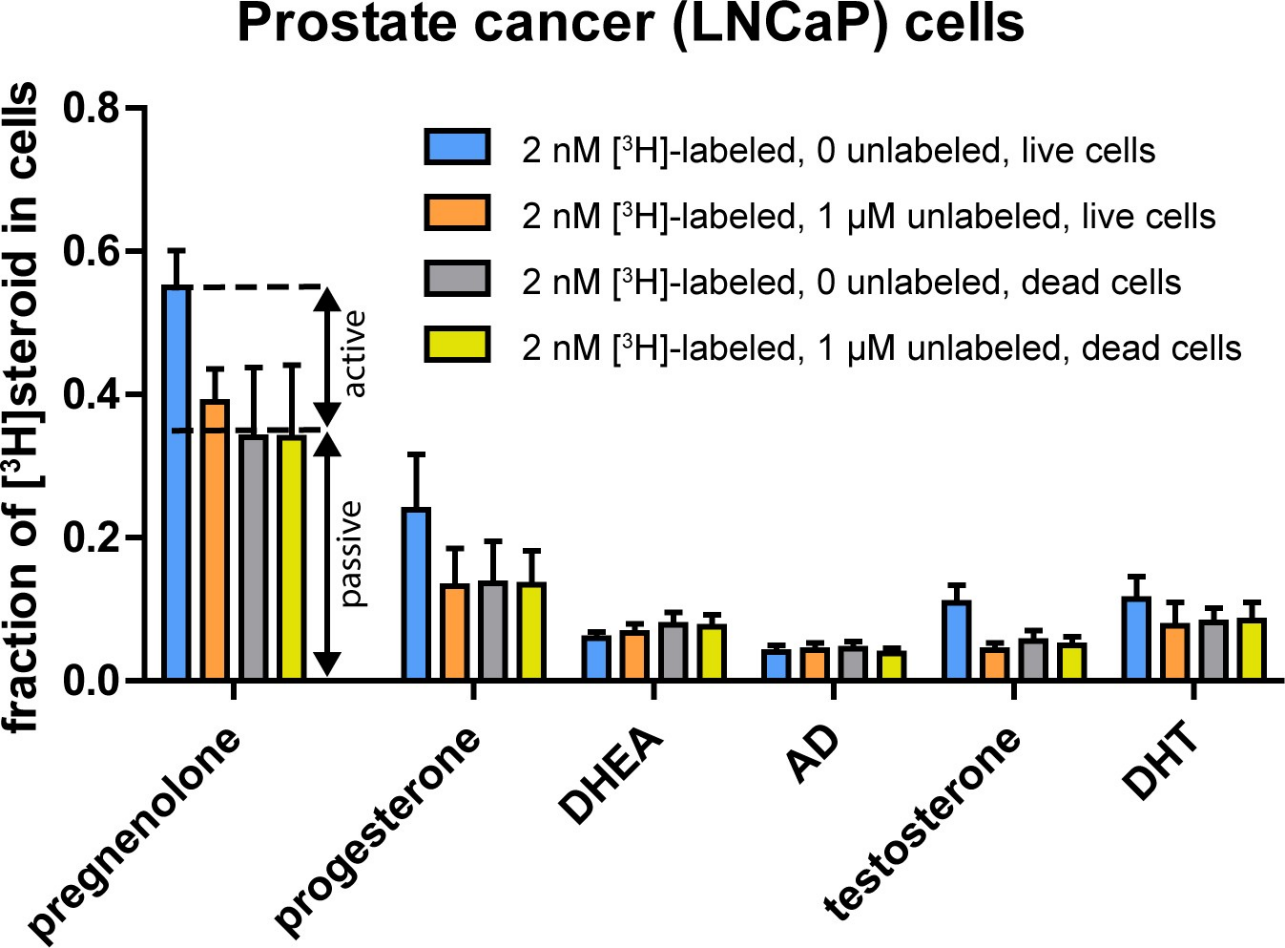
Live vs. dead cell uptake comparisons and uptake saturation both reveal contributions of active and passive accumulation. Fractions of total steroids (media plus cells) collected in the cell samples when live and dead LNCaP cells were incubated with 2 nM [^3^H]-labeled steroids alone and with 1 μM unlabeled steroids. For pregnenolone, progesterone, and testosterone, uptake of 2 nM [^3^H]-labeled steroid with 0 unlabeled steroid in live cells was greater than uptake in any of the other three conditions (Tukey’s multiple comparison test after one-way ANOVA, pregnenolone: p = 0.004 for blue vs. orange; p < 0.001 for blue vs. gray and blue vs. yellow, progesterone: p = 0.01 for blue vs. orange; p = 0.02 for blue vs. gray; p = 0.02 for blue vs. yellow, testosterone: p < 0.001 for all three comparisons). For DHT, p = 0.07 from one-way ANOVA.

To further test the hypothesis that elevated cellular concentrations of steroids are largely driven by passive mechanisms, we performed an additional experiment to test the contributions of passive vs. active uptake using two methods: the comparison of live vs. dead cell uptake described above, and a simultaneous test of the saturability of uptake. We reasoned that accumulation of [^3^H]-labeled steroid via active mechanisms (i.e., active transport across the plasma membrane, active binding to intracellular binding partners after passive diffusion across the membrane, or both) would be stopped either by heat-killing the cells prior to steroid incubation or by incubating the cells with an excess concentration of unlabeled steroid to saturate active transport and/or binding. We again treated cells with six steroids (pregnenolone, progesterone, DHEA, AD, testosterone, and DHT) under four simultaneous conditions: 2 nM [^3^H]-labeled steroid with no unlabeled steroid in live cells, 2 nM [^3^H]-labeled steroid with 1 μM unlabeled steroid in live cells, and the same two sets of concentrations in dead cells. The results (**Fig 3)** were suggestive of contributions of both active and passive accumulation in the prostate cancer (LNCaP) cells in which this experiment was performed. When active accumulation was not blocked (blue bars in **Fig 3**), pregnenolone, progesterone, and testosterone exhibited higher levels of steroid uptake than when active accumulation was blocked (orange, gray, and yellow bars in **Fig 3**); DHT also showed a trend toward higher uptake. By contrast, AD and DHEA exhibited no evidence of active accumulation. These findings are consistent with a report that active uptake by prostate cancer cells of testosterone is competed with by progesterone and DHT but not by DHEA [28]. The comparable passive uptake levels when active uptake was blocked by saturation (orange bars), by heat-killing cells (gray bars), or by both methods (yellow bars) support the viability of either method to differentiate passive from active accumulation. Although active mechanisms did contribute to the cellular uptake of several steroids, uptake of pregnenolone, the most highly taken up steroid, was mostly passive, and passive uptake of pregnenolone was greater than the total (active plus passive) uptake of any other tested steroid. Furthermore, the results support the conclusion that cellular preferences for progestogen and 3β-OH, Δ^5^-steroid uptake are largely driven by passive mechanisms.

### Half maximal free steroid uptake occurs within minutes with highest uptake favoring 3β-OH, Δ^5^-steroids and progestogens

**Fig 4:**
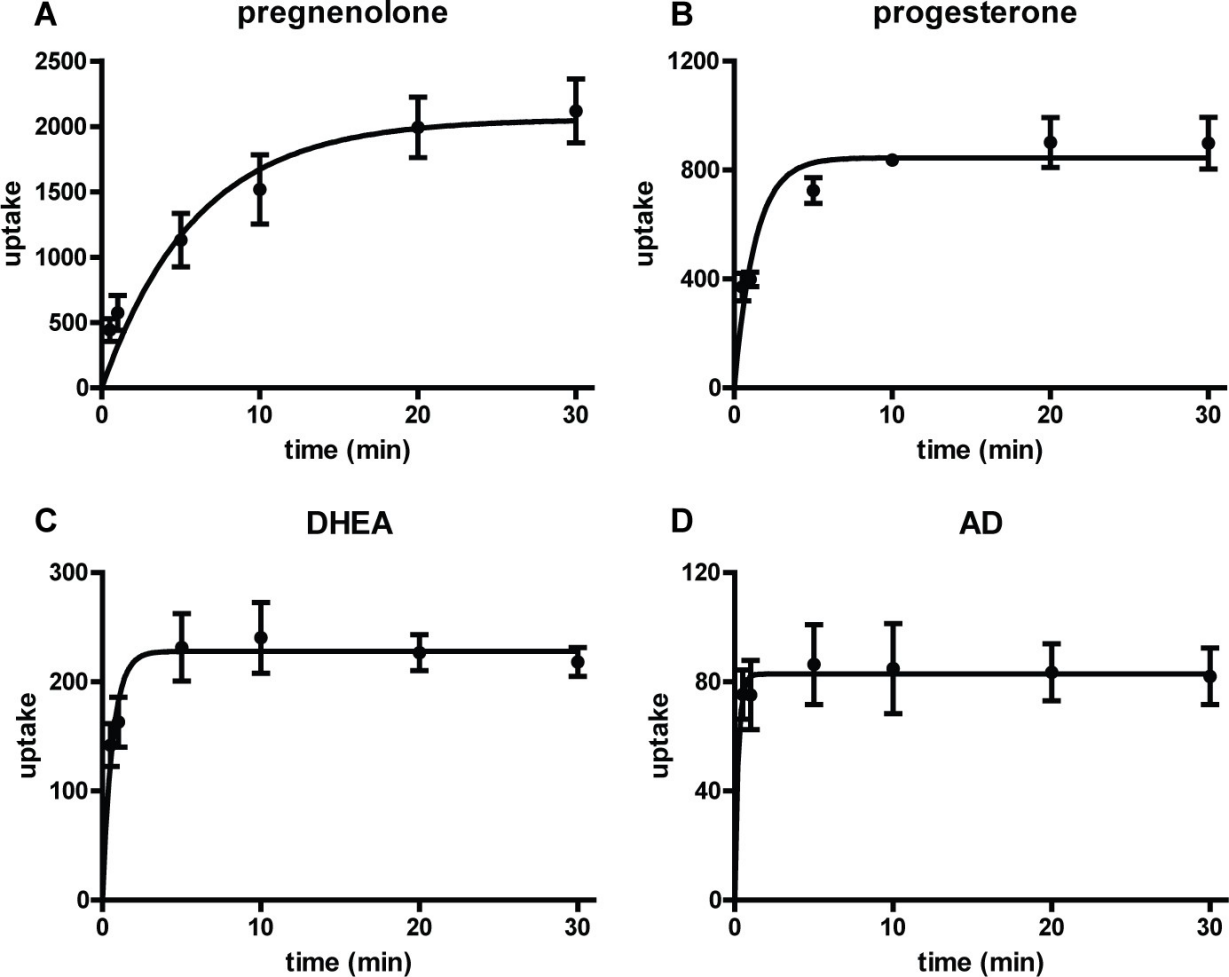
Half maximal free steroid uptake occurs within minutes with highest uptake favoring 3β-OH, Δ^5^-steroids and progestogens. Concentrations (mean ± SD) of pregnenolone **(A)**, progesterone **(B)**, DHEA **(C)**, and AD **(D)** in prostate cancer (LNCaP) cell samples at time points ranging from 0 to 30 min. Uptake is expressed in units of μL/mg (see Methods). One-phase association curves were fit to data using GraphPad Prism 5. Half-maximum times with 95% confidence intervals: pregnenolone 4.1 min (3.2–5.8 min), progesterone 0.91 min (0.75–1.18 min), DHEA 0.43 min (0.36–0.56 min), AD 0.16 min (0.10–0.35 min). The best-fit curve for each steroid was different from each other steroid’s best-fit curve (extra sum of squares F test, p < 0.001). All graphs represent three assays with duplicate samples; error bars represent standard deviations.

We next tested the time scale of cellular uptake for pregnenolone, progesterone, DHEA, and AD. Upon exposure to each of these steroids, cellular concentrations revealed rapid uptake with half-maximal uptake between 0.2–4.1 minutes. Notably, the time to reach equilibrium concentration increased in accordance with the cellular affinity for each steroid. In increasing order, AD, DHEA, progesterone, and pregnenolone had progressively higher half-maximum uptake times (**Fig 4**).

### Uptake of pregnenolone and conversion to progesterone are faster than uptake of DHEA and conversion to AD

**Fig 5:**
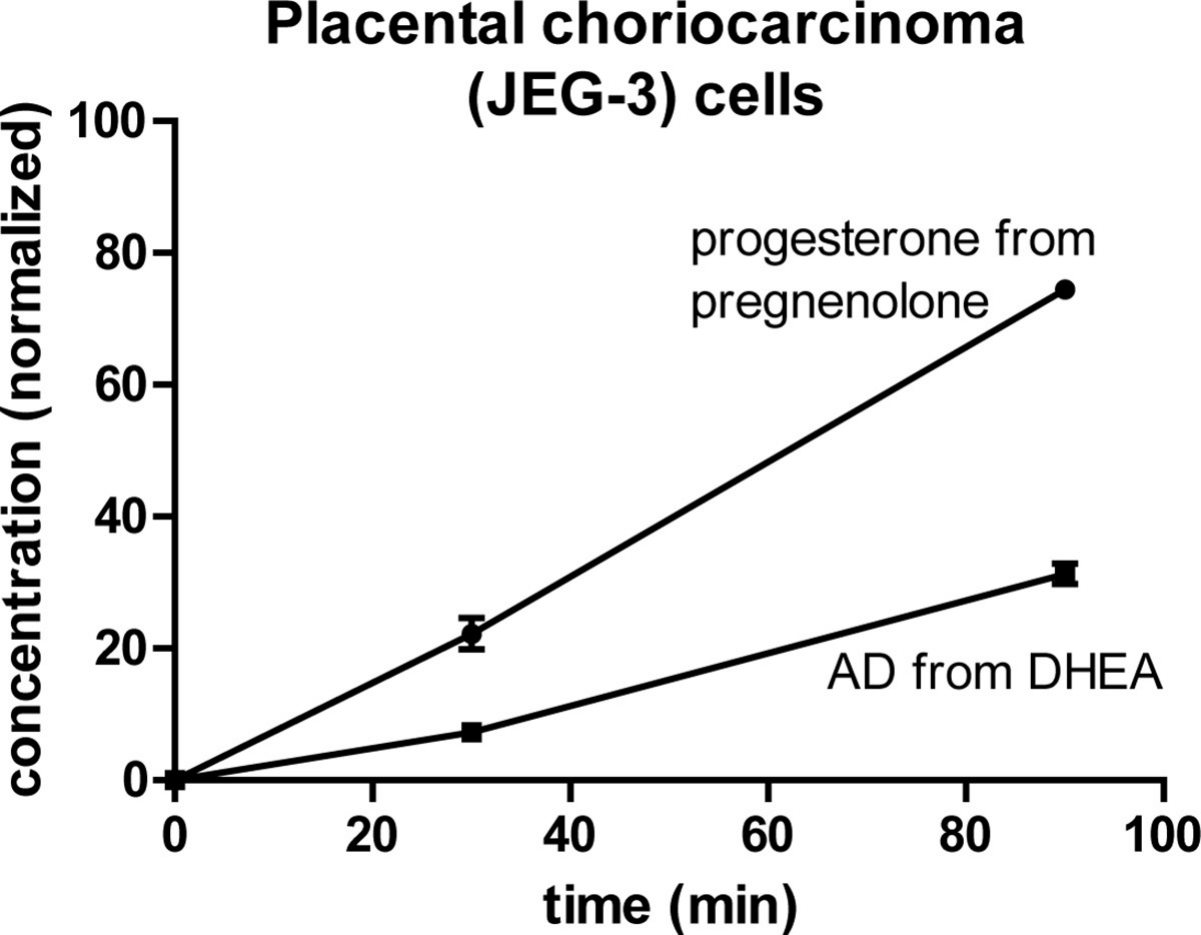
Uptake of pregnenolone and conversion to progesterone are faster than uptake of DHEA and conversion to AD. Normalized concentrations (mean ± SD) over time of progesterone and AD after JEG-3 cells were treated in parallel with 100 nM pregnenolone or DHEA. Results from one representative experiment with biological triplicates are shown; similar results were obtained in a second experiment.

The results thus far demonstrate that cells rapidly accumulate elevated concentrations of steroids in a structure-specific manner and utilizing largely passive mechanisms, but do not discriminate between the steroids entering the cells or simply binding to the cell surface or being incorporated into the plasma membrane. To help assess whether steroids are in fact reaching elevated concentrations within cells, we assayed enzymatic conversion of steroids incubated with cells, which requires the steroids to enter the cells before the reactions can occur. Placental cells express high levels of 3βHSD1, the enzyme that catalyzes conversion of 3β-OH, Δ^5^-steroids (e.g. pregnenolone and DHEA) to 3-keto, Δ^4^-steroids (e.g. progesterone and AD); the enzyme is localized to both mitochondria and endoplasmic reticulum [29]. We treated placental choriocarcinoma (JEG-3) cells separately with pregnenolone and DHEA and collected samples of cell culture media after 30 and 90 minutes. The results (**Fig 5**) are notable both for the rapidity of the reactions and the substantially faster conversion of pregnenolone than of DHEA. In an assay using purified enzyme, the kinetics of 3βHSD1-catalyzed conversions of pregnenolone and DHEA were reported to be very similar [29]. The faster reaction in cells that we observed of pregnenolone than of DHEA is consistent with a greater intracellular accumulation of pregnenolone. Furthermore, the finding that about half of the pregnenolone had already converted to progesterone within an hour is suggestive of a large pool of pregnenolone being inside the cells and available to the enzyme, as opposed to on the cell surface or in the plasma membrane. Taken in conjunction with our data on the time scale of uptake, these results suggest that large quantities of pregnenolone enter the cells within minutes, become available for reaction, and begin converting to progesterone. Then most of the progesterone exits the cells before being collected in the media samples we analyzed.

### Preferential accumulation of progestogens and 3β-OH, Δ^5^-steroids is observed in human prostate tissue

**Fig 6:**
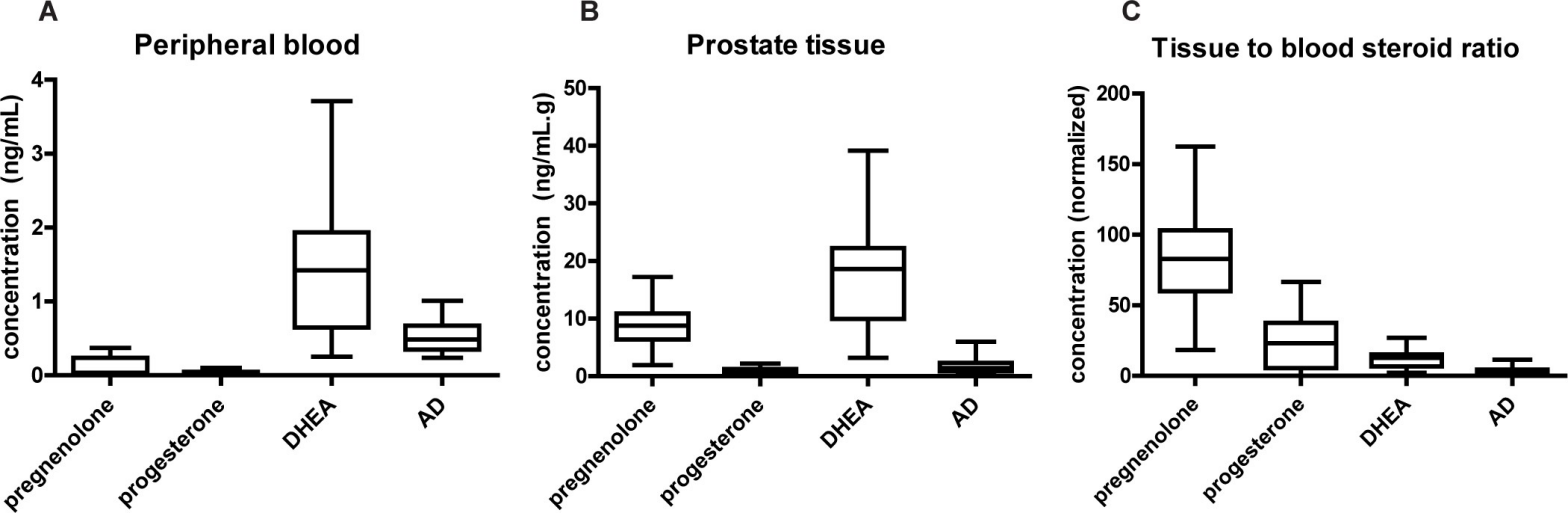
Preferential accumulation of progestogens and 3β-OH, Δ^5^-steroids is observed in human prostate tissue. Box and whisker plots of **(A)** blood concentrations, **(B)** normal prostate tissue concentrations, and **(C)** tissue to blood concentration ratios (tissue concentrations normalized to mean blood concentrations) for four steroids using samples from twenty prostate cancer patients. Prostatic tissue was collected at the same time as peripheral blood in patients undergoing radical prostatectomy. The ratio for pregnenolone was larger than the ratios for all other steroids; the ratio for progesterone was larger than the ratio for AD and the ratio for DHEA was larger than the ratio for AD (two-tailed Mann-Whitney U tests after Kruskal-Wallis test, p < 0.001 for all comparisons).

To explore the relevance of our findings on steroid uptake to human physiology, concentrations of pregnenolone, progesterone, DHEA, and AD were assessed by mass spectrometry in concomitantly collected peripheral blood and prostate tissue from twenty men who underwent radical prostatectomy (**Fig 6**). The mean (± SD) tissue concentrations normalized to mean blood concentrations were 85.2 ± 34.2 for pregnenolone, 23.1 ± 17.5 for progesterone, 12.3 ± 6.6 for DHEA, and 3.4 ± 2.5 for AD. Thus, all four steroids had higher concentrations in prostate tissue than in blood. Remarkably, these results support, in a human physiologic setting, the preferences for progestogens over androgens and 3β-OH, Δ^5^-steroids over 3-keto, Δ^4^-steroids to accumulate in tissue.

### Differences in octanol-water partition coefficients predict differences in cellular uptake of steroids

**Fig 7:**
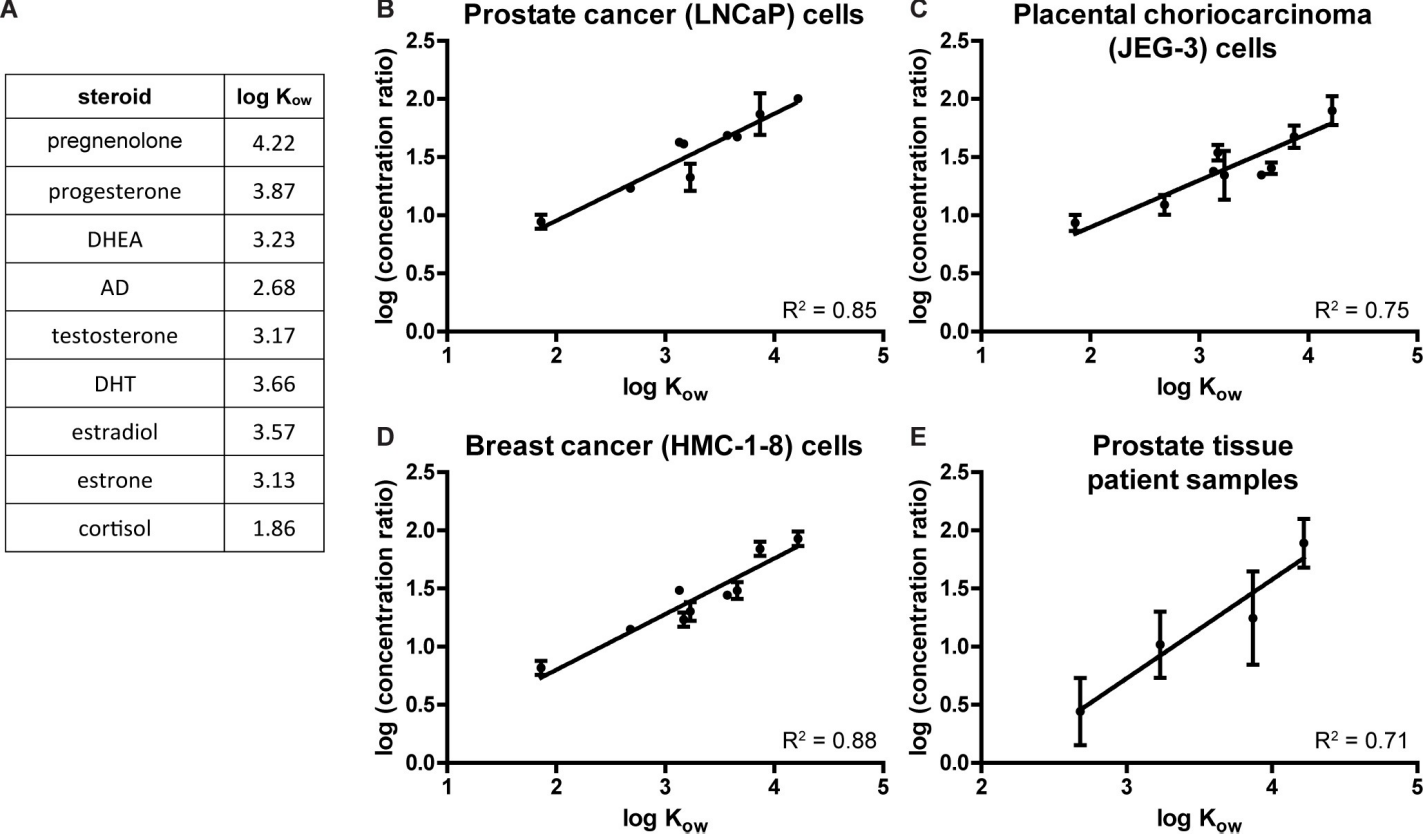
Differences in octanol-water partition coefficients predict differences in cellular uptake of steroids. **(A)** Experimentally determined log octanol-water partition coefficient (log K_ow_) values for nine steroids, obtained from Leszczynski and Schafer [30]. **(B-D)** Graphs of logs of average cell-to-media concentration ratios (from experiments described in **Fig 1**) vs. log K_ow_ for the same nine steroids, in **(B)** prostate cancer (LNCaP) cells, **(C)** placental choriocarcinoma (JEG-3) cells, and **(D)** breast cancer (HMC-1-8) cells. For each cell line, all steroids were assayed in at least two experiments with biological duplicates; graphs represent mean ± SD of concentration ratios. **(E)** Graph of logs of average tissue-to-blood concentration ratios (from data described in **Fig 4**) vs. log K_ow_ for pregnenolone, progesterone, DHEA, and AD. Data from twenty prostate cancer patients are included; graph shows mean ± SD of concentration ratios. For all graphs, slopes of trendlines are different from zero (p < 0.0001).

The octanol-water partition coefficient of a compound, usually expressed in log form (log K_ow_), measures the compound’s lipophilicity [25] and is known to predict the bioaccumulation of environmental toxins in aquatic organisms [18–20]. To test the predictive ability of log K_ow_ on cellular uptake of steroids in vitro and in vivo, we plotted the log of the cell-to-media or tissue-to-blood concentration ratio against the log K_ow_ of steroids. There are multiple sources from which both measured and predicted log K_ow_ values can be obtained; values from different sources do correlate with each other but not without inconsistencies. Therefore, to use one internally consistent set of measured log K_ow_s, values were obtained from a single study [30] in which the log K_ow_s of nine of the steroids we tested were experimentally determined (**Fig 7A**). The resulting correlations (**Fig 7B–7E**) show that lipophilicity measured by log K_ow_ strongly predicts the concentration ratios for different steroids in our in vitro results for cells derived from three different tissue types and in our in vivo results for peripheral blood and prostate tissue patient samples.

### More lipophilic steroids preferentially accumulate in membrane fractions of cells

**Fig 8:**
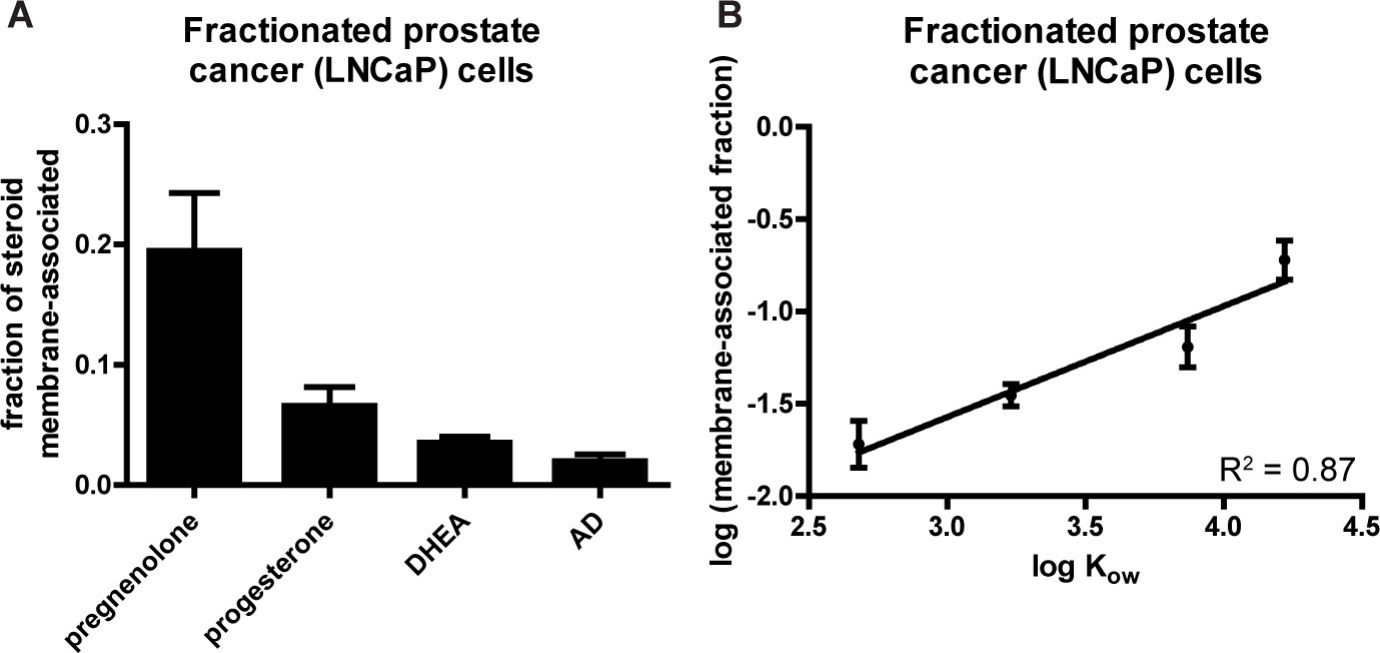
More lipophilic steroids preferentially accumulate in membrane fractions of cells. **(A)** Fraction of the total steroid content in the membrane plus cytosol fractions that was contained in the membrane fraction for four different steroids after fractionation of LNCaP cells that were treated with 1 μM unlabeled steroid. Graphs show mean ± SD from two independent experiments each performed in triplicate for each steroid. Pregnenolone content in membrane fractions was greater than any other steroid (Tukey’s multiple comparison test after one-way ANOVA, p < 0.001 for all three comparisons). **(B)** Graph of logs of the data from **(A)** vs. log K_ow_ of the four steroids. Slope of trendline is different from zero (p < 0.0001).

To directly test whether lipophilicity of steroids is correlated with steroid accumulation in lipophilic compartments of cells, we performed a fractionation experiment to isolate the membranes (plasma membranes and ER-Golgi membranes [24]) of LNCaP cells that had been treated with pregnenolone, progesterone, DHEA, or AD and measure the relative steroid content of the membrane and cytosol fractions. It is important to note that the results do not directly measure the steroid content of the membranes when they were contained in intact cells; free diffusion of steroid molecules when lysed cells are suspended in a volume of fractionation buffer that is much larger than the original volume of the cells makes it infeasible to do this measurement. However, the results do clearly show the same trends for membrane association of steroids as for overall cellular uptake of the same steroids, with pregnenolone having the most membrane association (**Fig 8A**), and the log of the steroid content in membrane fractions strongly correlating with log K_ow_ (**Fig 8B**)

### Modifying pregnenolone to decrease its lipophilicity results in decreased cellular uptake

**Fig 9:**
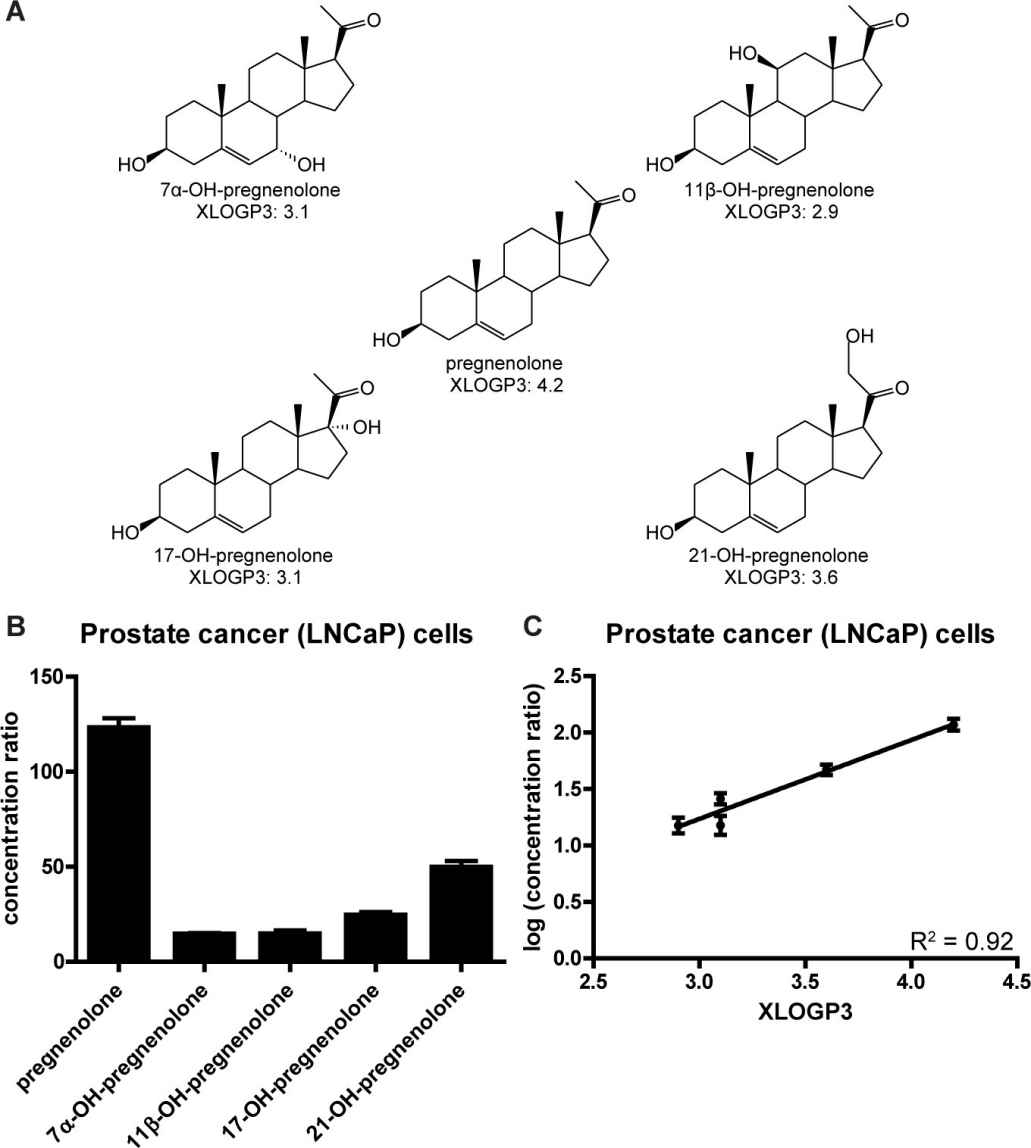
Modifying pregnenolone to decrease its lipophilicity results in decreased cellular uptake. **(A)** Structures of pregnenolone (center) and four different hydroxypregnenolones. Note the addition of a single -OH group at a different location on the structure of each hydroxypregnenolone. XLOGP3 values (computational predictions of log K_ow_s based on molecular structures) were obtained from PubChem. **(B)** Ratios of cellular concentrations to original treatment concentrations in culture media for the five steroids after treatment of LNCaP cells with 100 nM unlabeled steroid. Graph shows mean ± SD from one representative experiment with biological triplicates and the experiment was performed twice. The uptake of pregnenolone was greater than all other steroids (p < 0.001, Tukey’s multiple comparison test after one-way ANOVA). **(C)** Graph of logs of average cell-to-media concentration ratios vs. XLOGP3 for the five steroids. Graph shows mean ± SD from six total samples per steroid in two experiments. Slope of trendline is different from zero (p < 0.0001).

To further interrogate our finding that elevated cellular concentrations of steroids are largely driven by the steroid molecules’ lipophilicities, we performed an additional experiment to test the hypothesis that uptake of pregnenolone would decrease if pregnenolone was modified to decrease its lipophilicity. We predicted that hydroxylation would decrease the lipophilicity of pregnenolone, so we measured the uptake of pregnenolone in parallel to the uptake of four different hydroxypregnenolones; i.e., pregnenolones modified by addition of a single hydroxyl group (**Fig 9A**). XLOGP3 [31] values (computational predictions of log K_ow_s based on molecular structures) for these compounds show each is substantially less lipophilic than pregnenolone itself. Each of the hydroxypregnenolones was taken up less by cells than was pregnenolone (**Fig 9B**), and uptake strongly correlated with XLOGP3 (**Fig 9C**), lending further support to the conclusion that cellular uptake of free steroids is largely driven by lipophilicity.

### Serum proteins prevent much of the passive cellular uptake of steroids

**Fig 10:**
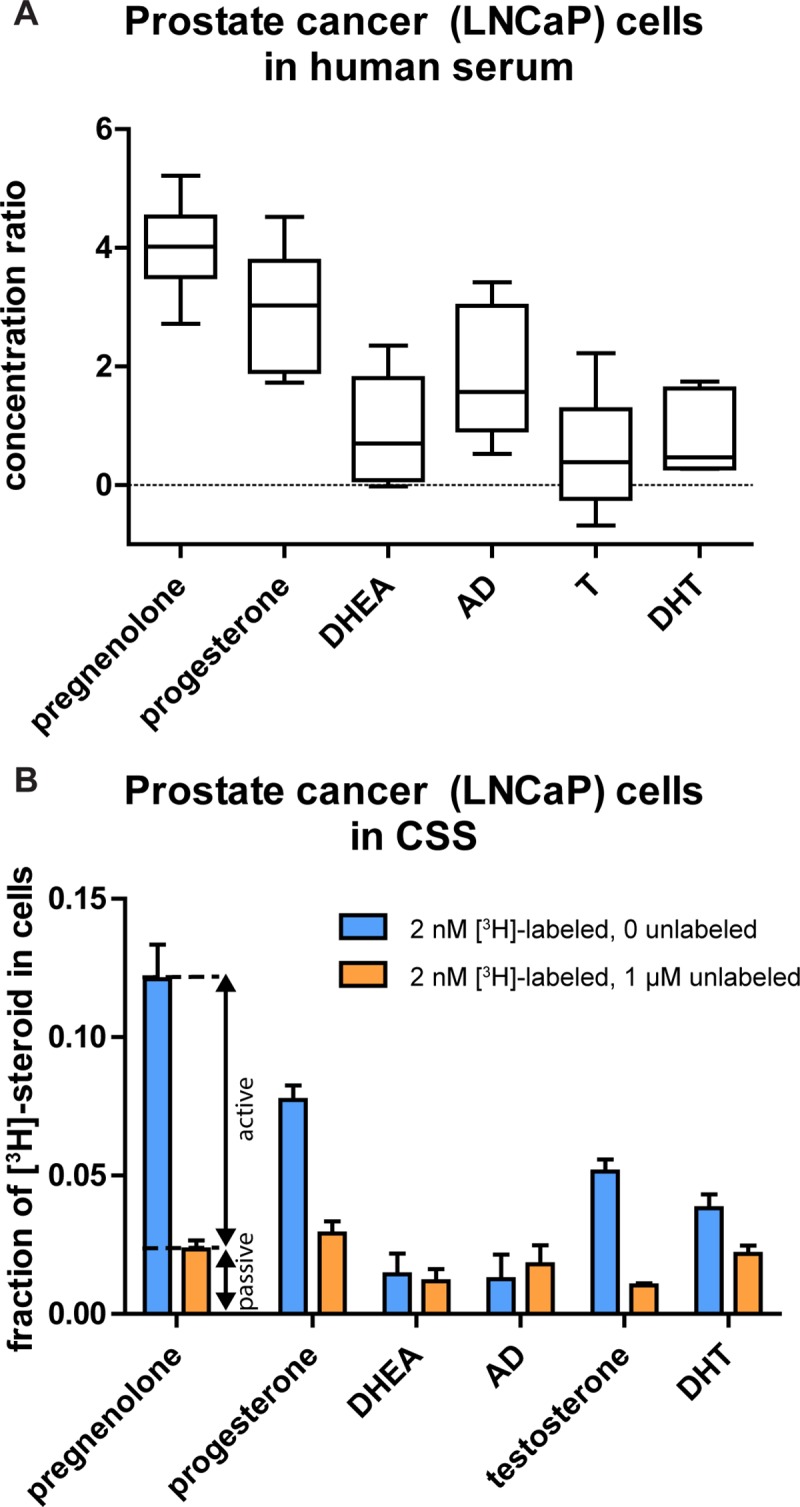
Serum proteins prevent most of the passive cellular uptake of steroids. **(A)** Box and whisker plot of concentration ratios for six steroids (2 nM [^3^H]-labeled steroid with 1 μM unlabeled steroid) in LNCaP cells in human serum. Experiments were performed in triplicate with at least two independent experiments for each steroid. Cellular steroid content was corrected by control experiments with no cells to account for effects of residual serum; note that this resulted in some values <0 for certain steroids, which is not physically possible, but these values were included to fully account for variability resulting from measurement uncertainty. Ratio for pregnenolone was greater than ratios for DHEA, AD, testosterone, and DHT (Tukey’s multiple comparison test after one-way ANOVA, p < 0.001 for all four). Ratio for progesterone was greater than ratios for DHEA (p = 0.003), testosterone (p < 0.001), and DHT (p = 0.002). For pregnenolone vs. progesterone, p = 0.17. **(B)** Fractions of total steroids (serum plus cells) collected in the cell samples when LNCaP cells were incubated in charcoal-stripped fetal bovine serum with 2 nM [^3^H]-labeled steroids alone and with 1 μM unlabeled steroids. Experiments were performed in duplicate with two independent experiments. Cellular steroid content was corrected by control experiments with no cells to account for effects of residual serum. For pregnenolone, progesterone, testosterone, and DHT, uptake of 2 nM [^3^H]-labeled steroid with 0 unlabeled steroid was greater than uptake with 1 μM unlabeled steroid (t-tests, p < 0.001 for pregnenolone, progesterone, and testosterone; p = 0.002 for DHT).

To directly test the capacity for cells to accumulate free steroids, all the in vitro experiments described thus far were performed using serum-free media, but in vivo a large proportion of steroid molecules are bound to serum proteins [32]. To test the effects of serum proteins on cellular steroid uptake, the uptake of pregnenolone, progesterone, DHEA, AD, testosterone, and DHT was tested in LNCaP cells with the culture media replaced with human serum prior to steroid treatment. In order to specifically test the effect on passive accumulation, a 2 nM [^3^H]-labeled steroid concentration with a 1 μM unlabeled steroid concentration was used, which our previous experiment (**Fig 3**) showed resulted in saturation of active accumulation. The results (**Fig 10A**) show that the cellular steroid concentrations were greatly diminished by the presence of serum proteins. Pregnenolone and progesterone still had heightened cellular concentrations but only in the two to fourfold range, whereas the other four steroids had concentration ratios close to one; i.e., the concentrations in cells and in serum were similar. We also examined the effect of serum proteins on the relative contributions of active vs. passive accumulation. We employed the design of 2 nM [^3^H]-labeled steroid both without and with 1 μM unlabeled steroid and performed the experiments with cells in charcoal-stripped fetal bovine serum so that endogenous steroids present in non-charcoal-stripped serum would not interfere with the uptake of [^3^H]-labeled steroid. The results (**Fig 10B**), as in our previous experiment with cells in serum-free media, showed that pregnenolone, progesterone, testosterone, and DHT appear to have both active and passive components of cellular uptake whereas DHEA and AD appear to only have passive components. Comparing **Fig 10B** to **Fig 3**, it can be seen that both passive and active accumulation were diminished by the presence of serum proteins, but the relative contribution of active mechanisms appears to be larger when serum proteins are present. For example, in **Fig 3** (no serum proteins present), about one-third of the 2 nM [^3^H]-labeled pregnenolone that accumulated in cells did so by active mechanisms; by contrast, in **Fig 10B** (serum proteins present), about 80% of the 2 nM [^3^H]-labeled pregnenolone that accumulated in cells did so by active mechanisms.

## Discussion

In this study, we have demonstrated that cells from multiple tissue types uniformly have a tendency to accumulate free (non-protein-bound) steroid molecules at elevated concentrations. Although active mechanisms appear to contribute to the uptake of certain steroids, blocking active mechanisms by heat-killing cells prior to steroid treatment or by saturation reveals strikingly large passive components of steroid uptake. All steroids we tested reach elevated cellular concentrations, but the degree to which this occurs is dependent on steroid molecular structure; for example, cells preferentially take up 3β-OH, Δ^5^-steroids over steroids with 3-keto, Δ^4^-structural features and progestogens over androgens. One caveat of the findings is that the initial sets of experiments (**Fig 1** and **Fig 2**) used [^3^H]-labeled steroid treatment amounts determined by activity, not by molarity, and therefore molar concentrations of different steroids were not exactly the same, but subsequent experiments utilized standardized molar concentrations of steroids and resulted in similar trends. The cellular accumulation of steroids occurs rapidly, within minutes, with a longer time course for steroids that reach higher concentrations. Rapid metabolism of pregnenolone by placental choriocarcinoma cells suggests that the cellular accumulation of steroids is not merely occurring at the cell surface or plasma membrane but in the interior of the cell. The structure-specific preferences that we observed in vitro persist in concomitantly obtained prostate tissue and peripheral blood patient samples, suggesting our findings may be relevant to human physiology. Our in vivo analysis surveyed a more limited set of steroids than our in vitro studies and only included one tissue type (prostate), a limitation that could be addressed by further studies. The degree of cellular steroid accumulation is predicted by the octanol-water partition coefficient of a steroid; more lipophilic steroids are taken up more by cells. More lipophilic steroids accumulate more in the membrane fraction of fractionated cells, and modifying a steroid to alter its lipophilicity correspondingly alters its cellular uptake, further supporting the hypothesis that cellular uptake of free steroids is largely driven by lipophilicity-based accumulation in lipophilic compartments of cells. Lastly, we showed that serum proteins prevent most of the passive uptake of steroids by cells, suggesting that passive cellular uptake of steroids may only draw from the free, not the protein-bound, steroid pool, while active uptake mechanisms may have some ability to draw from the protein-bound steroid pool.

The tendency of lipophilic molecules to accumulate in lipids (and, to a lesser extent, to adsorb to proteins) in a manner dependent on log K_ow_-measured lipophilicity has been known, in certain fields, for many years [25]. In that context, the main finding of our study, that free steroids accumulate in cells in a lipophilicity-dependent manner, is perhaps not, in a broad sense, surprising. However, to our knowledge, a systematic study of the lipophilicity-driven passive accumulation of differently structured steroids in cells has not been previously reported, and a literature review suggests that knowledge of the relevant concepts has not penetrated many of the fields to which they apply. Studies reporting heightened accumulation of steroids at the cell [33], in vivo human tissue [34], or intact whole animal [35, 36] level make no mention of lipophilicity-driven bioaccumulation, a concept that would provide important context for the results of such studies. Additionally, steroid receptor binding studies in which steroid concentration-dependent activation curves are generated typically account only for the steroid concentrations in media [37, 38] and not for the possibility that the concentrations in cells could be very different for differently structured steroids. Our finding that free steroids reach remarkably elevated cellular concentrations in a passive, structure-dependent manner is consistent with existing concepts of lipophilicity-driven bioaccumulation, but at the same time appears to be novel and important to fields such as steroid physiology in which these concepts have received little to no attention.

The apparent discrepancy between the elevated prostate tissue to peripheral blood concentration ratios we observed in vivo, an environment in which circulating steroids are mostly protein-bound, and the much less elevated ratios we observed when in vitro experiments were performed in serum is an issue that requires further study. Others have reported comparisons of serum and tissue concentrations for certain steroids and tissues with varying results. For example, two different groups [39, 40] reported elevated concentrations of DHEA and DHT in prostate tissue compared to serum, but testosterone concentrations that were greater in serum than in tissue. In vivo steroid concentration ratios are affected by factors other than cells’ tendency to take up steroids, including metabolism that occurs within cells; the depressed testosterone concentrations in prostate tissue could reflect its conversion to DHT by enzyme 5α-reductase [41]. In another study in which steroid levels in endometrial tissue were examined [42], healthy subjects did not have markedly elevated tissue-to-serum ratios of DHEA, AD, testosterone, pregnenolone, or progesterone. Particularly for the progestogens this contrasts notably with our findings in prostate tissue. One alternate explanation for the elevated concentrations we observed in prostate tissue could be production of progestogens and androgens from precursors pregnenolone sulfate and DHEA sulfate, both of which circulate at much higher concentrations than their unsulfated forms [43]. Addressing whether lipophilicity-driven passive accumulation is a major determinant of in vivo tissue concentrations of steroids would require a more systematic survey of different steroids and tissue types.

Interestingly, although heightened tissue concentrations have been cited as evidence for active uptake, it is rarely pointed out that, because most steroids circulate with >95% of molecules bound to serum proteins [32], if cells did not have any (active, passive, or both) means of accumulating steroids at heightened concentrations and uptake occurred via passive diffusion of free steroids, the total (free plus protein-bound) steroid concentrations in tissue could be expected to be much *lower* than the total steroid concentrations in blood. Given that cells *do* have a strong tendency to passively accumulate free steroids, as our results show, the binding of steroids to serum proteins may serve to ensure that a sufficient proportion of steroid molecules can circulate through the body rather than being caught in a sink of lipophilic compartments in cells and tissue, similar to a hypothesis put forth by Mendel et al. [44] regarding thyroid hormones.

Because steroid hormones must enter cells before carrying out most of their diverse actions, cellular uptake of steroids is relevant to many areas of physiology and disease, among them hormone-driven cancers such as those of the breast and prostate. Prostate cancer (PCa) growth is driven by testosterone and the more potent DHT activation of the AR. The mutant AR ligand binding domain (T877A) present in LNCaP cells and found in castration-resistant prostate cancer (CRPC) patients is strongly activated by additional ligands other than testosterone and DHT [45], including pregnenolone, which stimulates the growth of LNCaP cells [46, 47]. Thus the high capacity for cells to accumulate pregnenolone and the elevated pregnenolone concentrations we found in prostate tissue (**Fig 6**) may be of particular relevance to the effects of this and possibly other mutations [47]. Additionally, an association between progesterone receptor B expression and prostate cancer disease progression has been reported [48], further suggesting the potential relevance of elevated progestogen concentrations in prostate tissue.

Our findings have important implications for the design and interpretation of experiments in which cells are exposed to steroids in vitro. In such studies, cells are typically either in serum-free media or media with 10% serum. If a physiologic concentration of serum proteins were present, steroid uptake into cells, particularly passive uptake, would be greatly diminished (**Fig 10**). On the other hand, the rapid time course of diffusion, especially for less lipophilic steroids (**Fig 4**), could make it difficult to accurately measure the steroid content of cells, as a too thorough washing of cells could almost completely eliminate the passively generated cellular concentration of a less lipophilic steroid.

To our knowledge, the lipophilicity-driven tendency for cells to passively accumulate steroids in a structure-specific manner has not been previously described, but studies in several fields have provided evidence complementary to the findings we report here. Various studies have reported tendencies of aquatic organisms to accumulate heightened concentrations of steroids both in the wild [20, 49] and in controlled laboratory settings [35, 36, 50]. Cells in the aqueous environment of a tissue culture dish could be seen as analogous to organisms in the aqueous environment of a lake or stream. Within the body there are more forces at work than in vitro, including the presence of serum proteins that bind different steroids with different affinities, but our results suggest that cells in the body may accumulate steroids from the bloodstream in a similar manner to cells in a dish from their surrounding culture media. This tendency has not previously been recognized as a general property of cells, but other in vitro [51] and in vivo [34] studies have also shown examples of cells markedly accumulating certain steroids. Our results suggest that this is not just a specific property of certain cells, but rather a broad principle that arises from the physicochemical properties of cells and steroids.

A study by Chisari et al. of GABA_A_ receptor modulation by neuroactive steroids showed a striking relationship between steroid lipophilicity and modulatory potency; the cellular concentrations of the steroids were not measured, but the implication of the results is that more lipophilic steroids become more concentrated in the vicinity of the membrane-bound receptor [52]. The study also demonstrated, by fluorescence labeling, substantial accumulation of lipophilic steroids both in the plasma membrane and in intracellular compartments. This suggests a model in which lipophilic steroids bioaccumulate in all the lipophilic compartments of cells (i.e. the endoplasmic reticulum and membranes of organelles as well as the plasma membrane); our results are consistent with this model. In this model, diffusion-mediated steroid accumulation in lipophilic compartments within the cell’s interior could occur via passage through the endoplasmic reticulum, a continuous membrane network that is closely associated with the plasma membrane and the various organelles inside cells [53]. Intriguingly, although our results taken together with existing concepts of lipophilicity-driven bioaccumulation imply that most of the intracellular free molecules of a lipophilic steroid are contained in membrane structures, our results on pregnenolone metabolism in placental choriocarcinoma cells (**Fig 5**) imply the presence of a large pool of intracellular pregnenolone that is available as a substrate for enzyme 3βHSD1. This suggests the possibility that steroid molecules are continuously moving into and out of lipophilic compartments to re-equilibrate their concentrations as a reaction progresses.

Also consistent with our findings, in particular those on half-maximal uptake times (**Fig 4**), Chisari et al. showed that more lipophilic neuroactive steroids have longer time courses of modulatory effects and of cellular accumulation. Additionally, a study on transport of steroids across a Caco-2 cell monolayer showed that the transport occurred via passive diffusion and that there was a negative correlation between lipophilicity and the rate of diffusion across the monolayer [54]. These findings of correlations between lipophilicity and transit time could potentially factor into in vivo tissue to blood concentration ratios: when a steroid is produced within a tissue, a more lipophilic steroid could have a slower diffusion into circulation and therefore a greater tendency to be retained at elevated concentrations in the tissue.

Others have described evidence for active mechanisms mediating cellular steroid uptake [16, 17, 28, 55–57]. Our findings (**Fig 3, Fig 10**) provide additional evidence for active mechanisms, but suggest that these active mechanisms, which may be specific to certain steroids and types of cells and may include carrier-mediated transport processes as well as binding of steroids to nuclear receptors, occur in addition to the high capacity for passive uptake of free steroids that cells possess. Whereas elevated cell or tissue concentrations of steroids have been interpreted as evidence for active uptake [33, 34], our findings suggest that an elevated cell or tissue concentration is not in and of itself sufficient evidence for active mechanisms. The relative contributions of active vs. passive uptake are topics for further study; our data showing cells from different tissue types display similar but not identical uptake trends for different steroids (e.g. progesterone in **Fig 1E** vs. **1C** and **1D**) are suggestive that the differences may point to differential active uptake on top of a more uniform passive uptake background. In a study in *Drosophila* it was suggested that a membrane transporter is required for cellular steroid uptake and steroid hormones in *Drosophila* cannot enter cells via passive diffusion [16]. It is important to note that the steroid hormone being studied, ecdysone, has a relatively low lipophilicity (XLOGP3 = 1.7) and would therefore have a limited capacity to passively accumulate in cells. Additionally, the results of the study showed that knockdown of the relevant transporter substantially decreased but did not eliminate cellular uptake of ecdysone, and therefore do not rule out the presence of passive entry; our conclusion that a baseline level of passive steroid uptake is supplemented by active mechanisms for particular steroids and cell types is consistent with the results of this study. Taken together, the previous findings discussed here and the findings we report in this study suggest that structure-specific lipophilicity-driven steroid bioaccumulation is a general property of cells across tissue types.

## Supporting information

S1 Fig Mass spectrometry confirms steroid uptake in live and dead cells. Ratios of cellular concentrations to original treatment concentrations in culture media for three different steroids in live **(A)** and dead **(B)** LNCaP cells incubated in tubes and treated with 100 nM unlabeled steroid. All graphs show mean ± SD from one representative experiment with biological duplicates and all experiments were performed at least twice. For all graphs, the uptake of pregnenolone was significantly greater than all other steroids (p < 0.001, Tukey’s multiple comparison test after one-way ANOVA).
(TIF)

S2 Fig SHBG levels in patient blood samples. Box and whisker plot of sex hormone binding globulin (SHBG) concentrations in the peripheral blood patient samples used in **Fig 6**.
(TIF)
