# Supplementary figures and images for: Rapid and structure-specific cellular uptake of selected steroids
Source: PLoS One. 2019 Oct 17;14(10):e0224081. doi: 10.1371/journal.pone.0224081 (PMC6797172; doi:10.1371/journal.pone.0224081)

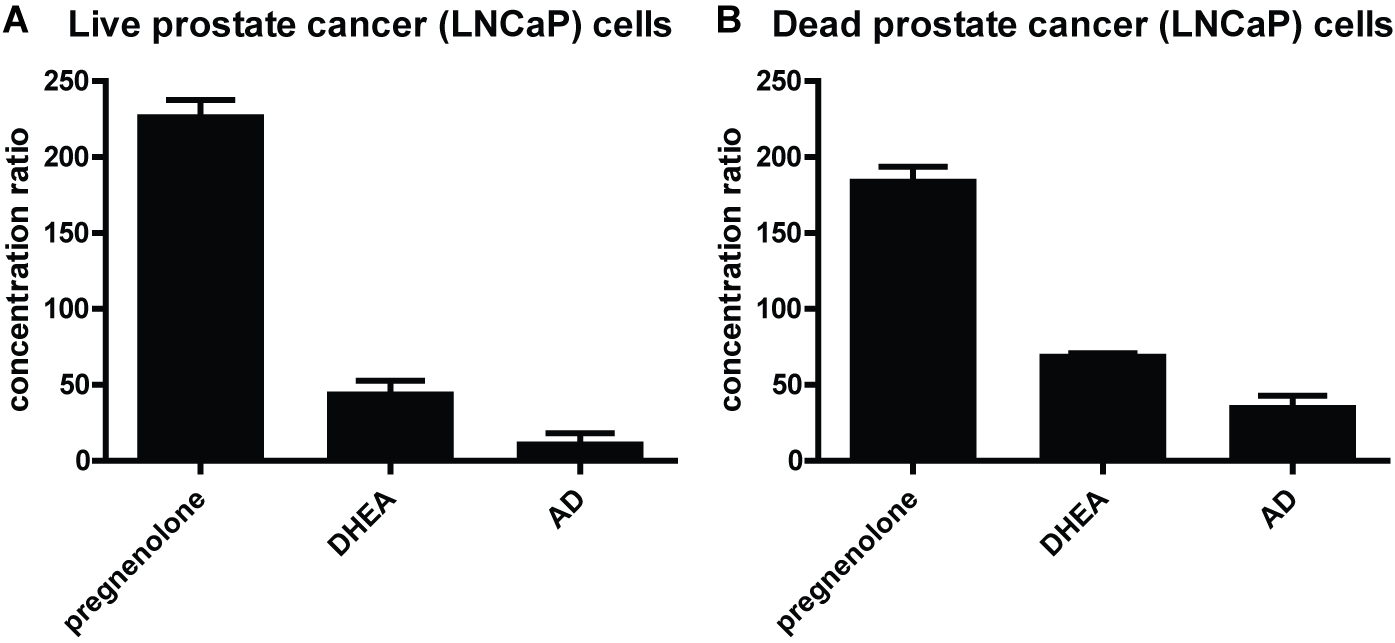

Supplement: S1 Fig — Ratios of cellular concentrations to original treatment concentrations in culture media for three different steroids in live (A) and dead (B) LNCaP cells incubated in tubes and treated with 100 nM unlabeled steroid. All graphs show mean ± SD from one representative experiment with biological duplicates and all experiments were performed at least twice. For all graphs, the uptake of pregnenolone was significantly greater than all other steroids (p < 0.001, Tukey’s multiple comparison test after one-way ANOVA). (TIF) [file pone.0224081.s001.tif]
